# Rickettsiosis Infections in Sarawak: Epidemiological Insights and Public Health Strategies

**DOI:** 10.1155/cjid/5218808

**Published:** 2025-09-10

**Authors:** Riz Anasthasia Alta Abang, Madinah Adrus

**Affiliations:** Faculty of Resource Science and Technology, Universiti Malaysia Sarawak, Kota Samarahan 94300, Sarawak, Malaysia

**Keywords:** and, Rickettsioses, risk factors, Sarawak, surveillance, transmission

## Abstract

Rickettsiosis infections pose a significant public health concern in Sarawak, Malaysia. Despite their importance, these infections remain poorly recognised, under-researched and inadequately documented within the context of the Sarawak region. This comprehensive evaluation seeks to address this knowledge gap by providing an in-depth analysis of Rickettsioses in Sarawak, focussing on four main aspects: the vectors and reservoirs of Rickettsioses, transmission dynamics and risk factors, existing challenges and public health implications and control strategies and future directions for raising awareness. Between 2013 and 2023, eleven Rickettsiosis cases were reported across seven districts in Sarawak. Three Rickettsial groups, namely, spotted fever group, typhus group and scrub typhus, have been identified in the region and linked to their respective arthropod vectors and vertebrate reservoirs. Although the overall number of confirmed Rickettsial infections in Sarawak remains relatively low, the detection of cases in both urban centres and smaller towns within rural areas raises concern regarding potential spread and under-recognition of these infections. This review underscores the urgent need to enhance regional awareness, strengthen disease surveillance and encourage focused research to bridge existing knowledge gaps. Developing evidence-based strategies for early detection, vector control and public health education will be crucial for preventing future outbreaks and safeguarding the health and well-being of communities throughout Sarawak.

## 1. Introduction

**Figure 1:**
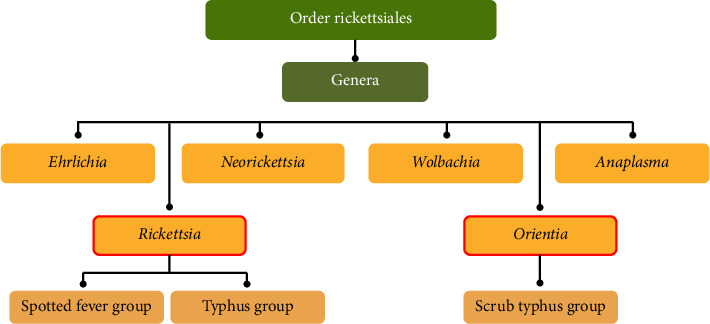
Genera under the order of Rickettsiales based on Yu and Walker's study [4].

Rickettsioses, also known as Rickettsial infections, are recognised as globally distributed diseases. They are caused by Gram-negative bacteria from the family *Rickettsiaceae*, which encompasses a diverse range of species within the order *Rickettsiales* and is considered to be continuously expanding. According to Fang et al. [1], there are currently 27 recognised species within the genus *Rickettsia*, approximately 17 of which have been identified as pathogenic to both humans and animals worldwide. Specifically, Rickettsioses are primarily caused by bacteria from the genera *Rickettsia* and *Orientia*, which are nonmotile, pleomorphic coccobacilli (Figure 1). These bacteria disseminate through the host's bloodstream and can significantly affect a range of primary cells and organs, particularly in human hosts. The principal clinical manifestations of Rickettsial syndromes in humans include fever, rash and eschar, often accompanied by other symptoms [2]. In recent years, intensive research has been devoted to Rickettsial infections, which have been reported to occur and expand globally. The geographic distribution of each species is closely associated with its arthropod vector, natural host and environmental conditions [3].

To date, fundamental studies on Rickettsioses in Sarawak remain limited, with only a few addressing Rickettsial infections, particularly in urban areas of East Malaysia. In recent years, several medical cases involving local patients hospitalised due to these infections have been documented. However, Rickettsial diseases continue to be neglected and are less well known compared to other established bacterial infections such as leptospirosis, as well as recent viral outbreaks in Sarawak, including rabies. Furthermore, Rickettsiosis has been recognised as endemic in Malaysia, particularly in rural regions [5, 6]. The relatively low annual incidence of Rickettsioses, coupled with the higher prevalence of other infectious diseases, has contributed to limited attention from institutions, healthcare professionals and the public. This is despite Rickettsioses being globally recognised as one of the most common emerging and re-emerging zoonotic diseases [7, 8].

Therefore, this paper objectively reviews and assesses the status of Rickettsioses in Sarawak and their implications for public health. It also presents the most recent data from the past decade regarding Rickettsia species, typhus infections, and cases of Rickettsioses in the region. Furthermore, this review discusses the common vectors and reservoirs of the infection, its transmission dynamics and associated risk factors, the challenges and public health implications and control strategies and future directions for raising awareness of Rickettsioses in Sarawak, Malaysia.

## 2. Methods

An extensive review was performed using scientific publications in English and Malay retrieved from databases such as Scopus, PubMed and Google Scholar. The search employed keywords including “Rickettsia”, “Rickettsioses”, “Rickettsial infections”, “Malaysia”, “Sarawak”, “Tick-borne disease”, “Mite-borne disease”, “Flea-borne disease” and “Typhus infections”. Publications were initially screened by titles and abstracts to identify those relevant to Rickettsial infections in Malaysia, before being narrowed to studies specific to East Malaysia (Sarawak and Sabah). All selected articles were thoroughly reviewed, and additional references cited within these papers, though not initially retrieved, were also examined. Statistical records and case data were obtained from healthcare professionals and the Sarawak State Health Department (JKNS) for inclusion in this review.

This review included a total of 20 relevant studies conducted across Malaysia. These comprised eight epidemiological or seroprevalence studies, four case reports and case studies, three studies focused on vector and reservoir identification, two molecular detection studies and three review papers. Among these, at least seven studies were conducted in East Malaysia (specifically in Sarawak and Sabah), while the remaining were based in Peninsular Malaysia. The seroprevalence studies primarily targeted high-risk populations such as indigenous communities, rural residents and military personnel. The case reports provided clinical insights into presentations of spotted fever, scrub typhus and murine typhus. Vector studies identified ticks, fleas and mites from rodents and domestic animals as carriers of Rickettsial pathogens. The molecular studies employed PCR-based detection techniques to identify *Rickettsia felis* and other species in arthropod vectors. Review papers offered a broader context on regional Rickettsial epidemiology and re-emergence trends. Overall, this distribution of the literature reflects an increasing but still limited research focus on Rickettsial infections in East Malaysia, underlining the need for further investigation using standardised molecular and serological diagnostic methods.

### 2.1. Vectors and Reservoirs of Rickettsioses in Malaysia

**Figure 2:**
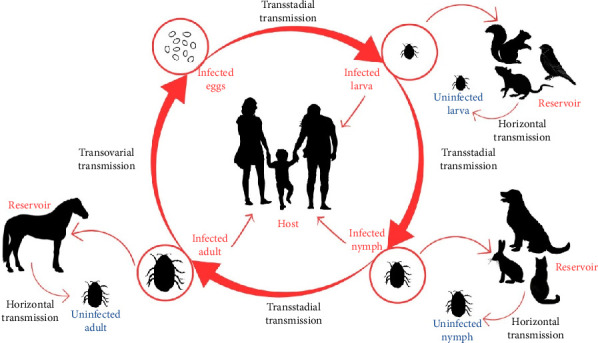
Transmission of Rickettsioses from different life stages of vectors and multiple hosts and reservoirs, adapted from [13].

The identification of vectors and reservoirs is crucial for elucidating the transmission dynamics of Rickettsial infections caused by various *Rickettsia* species. Ecologically, the life cycle of Rickettsiae is closely associated with arthropod vectors such as ticks, chiggers and fleas. These infections are primarily transmitted between hosts via either transovarial or horizontal transmission, with vertebrate hosts acting as reservoirs (Figure 2). Infection typically begins when an arthropod vector bites a vertebrate host to obtain blood or tissue fluid, subsequently releasing Rickettsiae through its saliva or faeces [9]. In addition, mosquitoes have also been identified as potential vectors for certain *Rickettsia* species in other regions of the world [10–12].

In Malaysia, two common genera of Rickettsial bacteria have been identified, namely, *Rickettsia* and *Orientia*, which belong to three principal groups of Rickettsiae: the spotted fever group (SFG), the typhus group (TG) and the scrub typhus group (STG) (Table 1). The SFG Rickettsiae are widely distributed across the Americas, Europe, Africa, Asia and Australia [14]. They are typically transmitted by ticks, mites and fleas, resulting in tick-borne, mite-borne and flea-borne Rickettsiosis [15–17]. Several case studies and clinical reports have indicated that SFG Rickettsial infections are often associated with travel and are commonly transmitted through bites and saliva during feeding activities [14].

The TG Rickettsiae are primarily transmitted through contact with infected rat fleas or the faeces of the human body louse [13, 18, 19]. These infections are typically found in urban and coastal regions characterised by dense rodent populations. In contrast, the STG Rickettsiae are strongly associated with chigger bites and are known to infest vertebrate hosts such as wild rodents and birds [20–24].

Moreover, in Malaysia, Rickettsial diseases are primarily transmitted through arthropod vectors such as ticks, mites, chiggers, lice and fleas [25–27] (Table 1). Recent studies in Malaysia have detected the Rickettsial pathogen *Rickettsia felis* in Laelapidae mites, representing the first such discovery in arthropod vectors within the country [27]. Additionally, ticks collected from small mammals have been examined for the presence of Rickettsial DNA; although some studies reported no positive samples, these findings underscore the need for broader and more systematic surveillance [25].

In Sarawak, Malaysia, *Rickettsia* species such as *Rickettsia rickettsii*, *R. typhi* and *Orientia tsutsugamushi* have been documented for decades. These pathogens were primarily detected in patients with acute infections during ecoepidemiological investigations [28]. With advancements in molecular diagnostics, the detection of *Rickettsia* in ecological research, particularly involving arthropod vectors such as ticks, mites and fleas, has significantly expanded. Ticks and rodents are regarded as the principal vectors and reservoirs of *Rickettsia* bacteria, which are responsible for Rickettsial diseases such as scrub typhus, SFG Rickettsioses, and murine typhus in Sarawak. A study conducted in Sarawak that screened engorged ticks collected from small mammals for *Rickettsia* DNA reported no positive detections. Researchers attributed this result to limitations in sample size or technical constraints, emphasising the need for continued and enhanced surveillance efforts [25]. Furthermore, a seropositivity analysis conducted from 2016 to 2020 revealed that Sarawak exhibited a higher rate of Rickettsial infections compared to neighbouring Sabah, with mites, fleas and ticks identified as the principal vectors of transmission [29].

### 2.2. Transmission Dynamics and Risk Factors of Rickettsioses in Sarawak

*Rickettsia* species have been identified on every continent except Antarctica, reflecting their global distribution. However, their presence in specific regions is often constrained by climatic conditions, vector availability and host specificity. Some *Rickettsiae*, such as *Rickettsia felis* and *Rickettsia typhi*, are globally distributed [30, 31]. Vector–host interactions provide critical insight into the transmission dynamics and risk factors of Rickettsioses in Sarawak. This is due to the complex relationships between hosts or reservoirs and their parasites, which are strongly influenced by the parasites' life cycles and modes of transmission. These dynamics are also shaped by the life history strategies, trade-offs, and ecological contexts of the host species [32–35]. Given the documented presence and distribution of Rickettsial reservoirs and vectors across Asia, it is anticipated that cases will continue to be reported in Sarawak over the next 10 to 15 years. This projection is supported by the current medical reports and epidemiological studies, which indicate a sustained risk of transmission in the region.

Ecologically, Rickettsioses are widespread in tropical and subtropical regions worldwide, where they disproportionately affect socioeconomically disadvantaged communities [8]. In assessing the distribution of this vector-borne disease across Southeast Asia, studies have identified scrub typhus and other Rickettsial infections as major causes of febrile illness in local communities in Thailand, Laos and Myanmar [36–39]. In the context of Malaysia, Yuhana et al. [19] reported that the transmission dynamics of Rickettsioses are shaped by geographical factors, with notable differences in disease patterns observed between the more urbanised regions of Peninsular Malaysia and the predominantly rural areas of East Malaysia.

Tick Typhus may be prevalent in remote interior villages in Sarawak due to increased contact with infected small wild rodents, which are recognised as primary vectors or hosts [36–39]. The risk of Rickettsioses in Sarawak is further elevated by its role as one of Malaysia's major ecotourism destinations. Several studies have reported that travellers engaging in outdoor activities such as jungle trekking, camping, hiking and rafting in rural settings face a heightened risk of exposure to Rickettsial infections [6]. In addition, other occupations and activities involving prolonged outdoor exposure, including agriculture, logging, land clearing, road construction and military operations, are considered significant risk factors for the transmission of Rickettsioses.

SFG Rickettsiae are predominantly harboured by ixodid ticks and are typically transmitted through bites and the injection of saliva during feeding [6, 26, 28, 40]. This transmission mechanism is also characteristic of most Rickettsiae carried by mites. In contrast, flea- and louse-borne Rickettsia can infect hosts through the introduction of faecal material into skin abrasions, cuts or bite wounds [41]. Martínez-Caballero et al. [42] reported that rocky mountain spotted fever (RMSF), caused by SFG Rickettsiae, involves transmission dynamics in which dogs and their associated ticks may play a key role, particularly in urban environments. In Sarawak, the emergence of rabies outbreaks associated with stray dogs, which are known to have a high lethality rate, has raised public health concerns. Consequently, stray dog populations in Sarawak may influence the transmission potential of RMSF, particularly in identified rabies hotspot areas.

Moreover, TG Rickettsial infections are typically associated with urban and coastal areas that harbour large rodent populations and are primarily transmitted through contact with the infected faeces of the human body louse [43]. Murine typhus, for example, occurs globally but is particularly prevalent in tropical and subtropical regions characterised by high human population density, close proximity to rodents and poor sanitation. These environmental and social conditions are recognised as potential hotspots for outbreaks, especially within the context of Sarawak [5, 44–47]. Several studies have also demonstrated that infection with TG Rickettsiae may occur via inhalation through the aerosolisation or contamination of airborne dust particles [46]. Rare cases of transmission have been reported through conjunctival exposure to contaminated air and environmental surfaces. In Sarawak, the only recorded TG Rickettsial infection is murine typhus, caused by *Rickettsia typhi* (Table 1).

**Figure 3:**
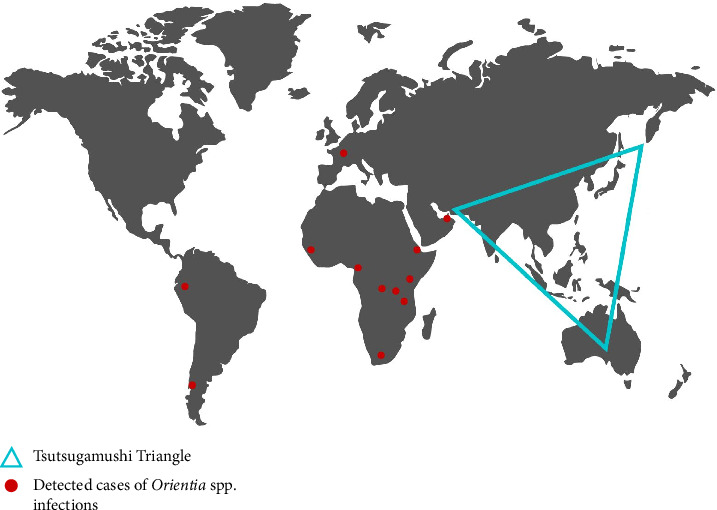
Human and animal serological and molecular evidence of *Orientia* spp. infections in new endemic regions of scrub typhus outside of the Tsutsugamushi Triangle, adapted from Jiang and Richards's study [52].

One of the known endemic Rickettsial infections in Asia, Australia and the islands of the Indian and Pacific Oceans is STG Rickettsial infection, represented by *Orientia tsutsugamushi* [48–51]. As illustrated in Figure 3, the geographical distribution of scrub typhus is referred to as the Tsutsugamushi Triangle, which spans from northern Japan and eastern Russia in the north to northern Australia in the south and Pakistan in the west [8]. In Sarawak, *O. tsutsugamushi* was documented in multiple studies as early as the 1920s (see Table 1), aligning with Malaysia's inclusion within the Tsutsugamushi Triangle. Nevertheless, certain regions were traditionally regarded as free from scrub typhus until recent investigations challenged this assumption. Jiang and Richards [52] reported growing evidence of STG Rickettsial infections emerging beyond the boundaries of the Tsutsugamushi Triangle. With the continuing impact of climate change and rising global temperatures, it is expected that many Rickettsial diseases currently confined to tropical regions will extend their geographical range.

### 2.3. Challenges and Public Health Implications of Rickettsioses in Sarawak

One way to understand the challenges in identifying and recognising Rickettsioses in Sarawak is by examining the lack of recognition of Rickettsial diseases as significantly neglected tropical diseases in Malaysia. In Sarawak, public health efforts are predominantly focused on other animal-borne and vector-borne diseases such as rabies, leptospirosis, malaria, and Japanese encephalitis (JE). The exclusion of Rickettsioses from the list of neglected tropical diseases has considerable implications, particularly for the training of local clinicians. Medical trainees are often led to believe that Rickettsial infections are rare or even unlikely to occur in Sarawak, thereby reducing clinical suspicion during the diagnosis.

This misperception can result in a systematic bias against considering Rickettsial pathogens as possible aetiological agents of febrile illness in clinical settings. Consequently, the lack of diagnostic infrastructure and general awareness can lead to delays or failure in treatment, increasing the risk of morbidity and mortality. Moreover, it represents a missed opportunity for cost-effective interventions and targeted prevention strategies aimed at controlling vectors and zoonotic reservoirs. Perhaps most concerning is that underrecognition can lead to misdiagnosis and the misuse of antibiotics, further exacerbating the global challenge of antimicrobial resistance. From a research perspective, this lack of awareness has also contributed to limited funding opportunities, thereby discouraging early-career scientists from engaging in the field. This, in turn, restricts advancements in translational research, including the development of diagnostics, vaccines and therapeutics.

Based on the data obtained from the Sarawak State Health Department (JKNS) over a 10-year period from 2013 to September 2023 (Table 2), five years were identified as Rickettsioses-affected: 2015, 2018, 2019, 2020 and 2021. The highest number of cases was reported in 2019, accounting for six out of a total of 11 detected cases (54.5%). Eleven confirmed cases of Rickettsioses in Sarawak were diagnosed following standard protocols used in Malaysia's public healthcare system. Typically, patients with suspected symptoms identified at local hospitals were referred to the Institute for Medical Research (IMR) for confirmatory serological testing. Most often, with the use of indirect immunofluorescence assay (IFA) or enzyme-linked immunosorbent assay (ELISA).

In recent years, some healthcare facilities in Sarawak have also adopted commercially available rapid tests, such as the Rapid Rickettsia IgG/IgM Combo Test Card. This medical technique allows for earlier detection at the point of care. These diagnostic approaches are in line with the national guidelines for vector-borne disease surveillance. Due to privacy regulations, detailed patient histories are not available for all cases; however, the confirmed reports represent genuine, laboratory-confirmed infections and provide important insights into potential exposure risks in both urban and rural areas of Sarawak. Although the overall number of reported cases remains low and does not reflect the true scale of infection among the population in Sarawak, it is important to consider that these figures may be underestimated due to unreported or unpublished data. Despite the limitations, the existing data remain valid and highlight the need for improvement in disease surveillance, reporting and diagnostic practices.

**Figure 4:**
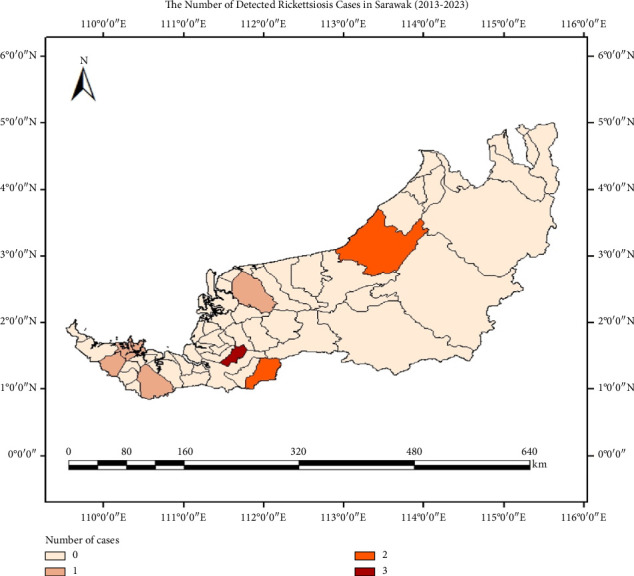
Data summary based on the number of Rickettsiosis cases in Sarawak for a 10-years range.

Figure 4 illustrates that out of the 32 districts in Sarawak, seven were recorded as Rickettsiosis-affected districts. These seven districts are primarily urban areas, with the exception of Lubok Antu and Bau, which are small towns located in rural regions. Overcrowded urban settings are more likely to report cases of Rickettsioses, as these areas are associated with a higher risk of outbreaks due to increased interactions with small mammals such as rodents, stray dogs and cats. These animals thrive in human-modified environments and are recognised reservoirs of various zoonotic pathogens that pose a risk to public health.

Children represent another vulnerable group due to their frequent exposure to specific vectors, including brown dog ticks and fleas from cats or rats [53, 54]. In recent years, rats and other rodents have been identified as the primary vertebrate hosts for Rickettsial infections, particularly those caused by SFG Rickettsia [55, 56]. Since the main vectors of Rickettsial infections feed on rodents, human populations living near rodent-infested areas face an increased risk of exposure.

From a socioeconomic perspective, Sarawak is a developing state in Malaysia, renowned for its rich biodiversity. Rural and small-town communities, many of whom depend on farming and small-scale agriculture, are particularly vulnerable to Rickettsial infections. Prolonged outdoor activities in agricultural settings increase their risk of exposure to infected animal reservoirs and vectors. Furthermore, substantial evidence suggests that populations living in poverty and with limited access to modern healthcare are disproportionately at risk of infection and vector exposure [57].

Although the number of reported Rickettsial disease cases in Sarawak between 2013 and 2023 remains low, this figure likely does not reflect the true burden of disease in the region. Given Sarawak's large geographic area, widespread rural population and ecological conditions favourable for vector-borne transmission, the low case numbers may be attributed to underreporting, limited diagnostic capacity and gaps in disease surveillance, particularly in remote or underserved areas. Therefore, it is essential to enhance the disease surveillance system, expand access to diagnostics and raise awareness among the public and healthcare professionals. These measures and actions are crucial for ensuring early detection, accurate reporting and effective management of Rickettsial infections throughout Sarawak.

### 2.4. Recommended Framework of Integrated Control Strategies for Rickettsioses in Sarawak

To date, there are no documented control strategies or vector management programmes specifically aimed at preventing Rickettsial infections in Sarawak. While the number of reported cases remains relatively low, the detection of *Rickettsia* spp. in human, animal and vector populations highlights the potential for these infections to escalate if left unaddressed. In light of this, the following section outlines a recommended framework for control strategies to enhance awareness, surveillance and prevention of Rickettsioses in Sarawak.

**Figure 5:**
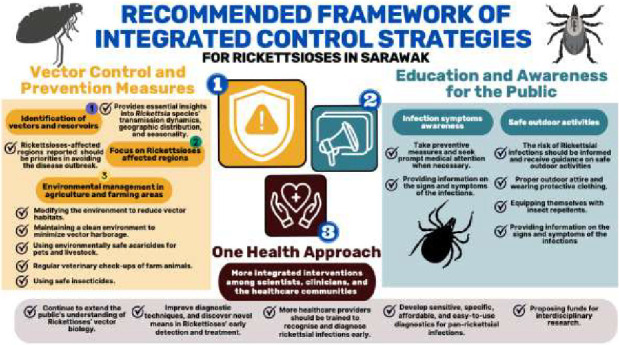
Recommended framework of integrated control strategies of Rickettsioses in Sarawak.

Numerous general preventive measures can be implemented to address arthropod-borne diseases, especially Rickettsioses. These strategies include vector control and prevention efforts, public education and awareness initiatives and the One Health approach, which promotes a more integrated intervention among scientists, clinicians and healthcare communities (Figure 5).

Identifying vectors and reservoirs is a critical component of Rickettsial infection research, as it offers valuable insights into the transmission dynamics, geographic distribution and seasonal patterns of *Rickettsia* species. Implementing vector control measures is particularly important in endemic regions. In Sarawak, areas affected by Rickettsioses should be prioritised to prevent disease outbreaks. Control measures in agricultural and farming areas should focus on reducing vector and reservoir populations through environmental management, the use of environmentally safe acaricides for pets and livestock and maintaining a clean environment to minimise habitats for ticks, mites and fleas. These strategies are essential for limiting the spread of Rickettsial agents via their arthropod vectors. The development and implementation of strategies for modifying the environment and reducing vector habitats is crucial, particularly in areas with high tick or flea prevalence, such as rodent-nesting sites, which can help disrupt the transmission cycle and lower the risk of Rickettsioses. Additionally, safe handling of pets should be highlighted. Pet owners need to be informed about the risk of infection and regular veterinary check-ups, along with the use of safe insecticides, contribute to the overall prevention of Rickettsioses in both humans and animals.

Since Sarawak is renowned for its ecotourism attractions, it is essential to enhance education and awareness regarding safe outdoor activities. Both travellers and local residents should be informed about the risks associated with Rickettsial infections and provided with guidance on how to engage in such activities safely. This includes disseminating information on the signs and symptoms of these infections, which can empower individuals to take preventive measures and seek timely medical attention when necessary. Outdoor activities, particularly in areas with a high risk of tick and flea exposure, should be undertaken with appropriate precautions. Individuals are advised to wear protective clothing such as gloves, long-sleeved shirts, long trousers and closed-toe footwear. Furthermore, carrying insect repellents and conducting routine tick checks after time spent in wooded or grassy areas are essential steps for the early identification and removal of ticks. These precautions are equally applicable to those employed in high-risk occupations, such as forestry, agriculture or wildlife management.

In addition to developing vector-targeted control strategies to safeguard public health, researchers and public health professionals must continue to strengthen public understanding of the vector biology associated with Rickettsioses, enhance diagnostic capabilities and explore innovative approaches for the early detection and treatment of these infections. Members of the public are encouraged to seek medical attention promptly if they experience symptoms such as fever, rash or influenza-like illness following potential exposure to arthropod vectors. Early detection is vital for effective treatment. Therefore, healthcare providers, particularly in regions of Sarawak affected by Rickettsial infections, should receive appropriate training to recognise and diagnose such infections at an early stage in order to initiate timely and effective treatment.

Due to its complex ecology, Rickettsioses prevention necessitates a One Health approach [8]. There is also a pressing need to expand ecoepidemiological studies on Rickettsioses in Sarawak. This includes fostering greater communication and collaboration among scientists from various disciplines to establish community-based surveillance programmes. The implementation of robust reporting systems for suspected cases can facilitate rapid response measures, enable monitoring of vector populations and human cases and help prevent the further spread of Rickettsial infections within the affected communities. Furthermore, the Sarawak State Government could take the initiative to allocate funding for interdisciplinary research aimed at developing sensitive, specific, affordable and user-friendly diagnostics for pan-Rickettsial infections. Supporting the research and development of vaccines targeting specific Rickettsial species is also crucial. In areas with a high incidence of Rickettsioses, or those identified as potential outbreak zones, the development of effective vaccines may enhance community-level immunity and reduce the severity and incidence of infections.

## 3. Conclusion

In conclusion, this comprehensive investigation of Rickettsioses in Sarawak has provided valuable insights into various aspects of this often overlooked public health issue. The review identifies four key areas that contribute to a better understanding of the region's epidemiology and the management of Rickettsioses. However, ongoing challenges such as limited resources, inadequate healthcare infrastructure and a general lack of public awareness continue to impede progress in controlling the disease. Despite improvements in understanding, significant knowledge gaps remain, emphasising the urgent need for further research.

The identification of vectors and reservoirs is essential for implementing effective control measures. It is crucial to develop strategies that include integrated vector management, public health education, long-term surveillance programmes and advanced diagnostic tools in order to reduce the prevalence and impact of Rickettsioses in Sarawak. A clear understanding of transmission dynamics is vital for designing targeted interventions, while recognising specific risk factors can help to inform surveillance and prevention strategies. Collaboration among healthcare professionals is important, and addressing the challenges posed by Rickettsioses in Sarawak requires a concerted effort from all sectors of the community. Such collective action is a key to strengthening preparedness and improving the response to Rickettsial infections, ultimately safeguarding the health and well-being of the population.

## Figures and Tables

**Table 1:** Three major Rickettsial groups have been identified in Malaysia.

Rickettsial group	Bacteria species	Disease	Primary arthropod vector	Vertebrate reservoir/host	References
Spotted fever group (SFG)	^*∗*^ * Rickettsia rickettsii*	Rocky mountain spotted fever	Ticks	Rodents, dogs	[26], [28]^∗^
	*Rickettsia conorii* subsp. *indica*	Boutonneuse fever/Indian tick typhus	Ticks	Rodents, dogs	[6], [40]
	*Rickettsia honei*	Thai tick typhus	Ticks	Rodents, dogs, wild mammals, reptiles	[6], [15], [17]
	*Rickettsia felis*	Cat-fleas typhus	Cat fleas, mites	Humans, domestic cats, opossums, rodents	[27], [40]
	*Rickettsia raoultii*	Tick-borne lymphadenopathy (TIBOLA)	Ticks	Rodents	[26]
	*Rickettsia australis*	Queensland tick typhus	Ticks	Rodents	[6],

Typhus group (TG)	*Rickettsia prowazekii*	Epidemic typhus/Brill–Zinsser disease	Human body louse, flying squirrel ectoparasites	Humans, rodents, flying squirrel	[43]
	^*∗*^ * Rickettsia typhi*	Endemic or murine typhus	Rat fleas	Rodents	[5], [17], [19], [24], [26], [28]^∗^, [43], [45], [47]^∗^

Scrub typhus group (STG)	^*∗*^ * Orientia tsutsugamushi*	Scrub typhus	Larval stage *Leptotrombidium* mites (chiggers)	Humans, rodents	[5], [15], [19–24], [27], [28]^∗^, [47]^∗^

^∗^Vector species and references for Rickettsial infections recorded in Sarawak.

**Table 2:** The number of detected Rickettsioses cases in Sarawak in 10 years (2013–September 2023), based on the affected districts and year acquired from the Sarawak State Health Department (JKNS) and the rate of cases per year.

Year	District	Number of cases	Rate of cases (%)
2015	Kuching	1	9.0

2018	Bau	1	18.2
	Sibu	1	

2019	Lubok antu	2	54.5
	Betong	3	
	Bintulu	1	

2020	Bintulu	1	9.0

2021	Serian	1	9.0

Total number of cases		11 cases	

Total district cases		7 districts	

## Data Availability

The data that support the findings of this study are openly available in [repository name] at [DOI].
